# Plasma HIV-1 Tropism and the Risk of Short-Term Clinical Progression to AIDS or Death

**DOI:** 10.1371/journal.pone.0166613

**Published:** 2017-01-27

**Authors:** Maria Casadellà, Alessandro Cozzi-Lepri, Andrew Phillips, Marc Noguera-Julian, Markus Bickel, Dalibor Sedlacek, Kai Zilmer, Bonaventura Clotet, Jens D. Lundgren, Roger Paredes, EuroSIDA in EuroCOORD

**Affiliations:** 1 IrsiCaixa AIDS Research Institute, Badalona, Catalonia, Spain; 2 Universitat Autònoma de Barcelona, Catalonia, Spain; 3 Royal Free Hospital, London, United Kingdom; 4 Universitat de Vic-Universitat Central de Catalunya, Vic, Catalonia, Spain; 5 Goethe University, Frankfurt/Main, Germany; 6 Charles University Hospital, Plzen, Česká Republika; 7 West-Tallinn Central Hospital, Tallinn, Estonia; 8 HIV Unit, Hospital Universitari Germans Trias i Pujol, Badalona, Catalonia, Spain; 9 CHIP, Department of Infectious Diseases, Rigshospitalet, University of Copenhagen, Copenhagen, Denmark; National and Kapodistrian University of Athens, GREECE

## Abstract

**Objective:**

To investigate if plasma HIV-1 tropism testing could identify subjects at higher risk for clinical progression and death in routine clinical management.

**Design:**

Nested case-control study within the EuroSIDA cohort.

**Methods:**

Cases were subjects with AIDS or who died from any cause, with a plasma sample with HIV-1 RNA >1000 copies/mL available for tropism testing 3 to 12 months prior to the event. At least 1 control matched for age, HIV-1 RNA and HCV status at the time of sampling were selected per each case. Conditional logistic regression was used to investigate exposures associated with clinical progression to AIDS or death. A linear mixed model with random intercept was used to compare CD4^+^T-cell slopes by HIV tropism over the 12 months following the date of sampling.

**Results:**

The study included 266 subjects, 100 cases and 166 controls; one quarter had X4 HIV; 26% were ART-naïve. Baseline factors independently associated with clinical progression or death were female gender (OR = 2.13 vs. male, 95CI = 1.04, 4.36), p = 0.038), CD4^+^T-cell count (OR = 0.90 (95CI = 0.80, 1.00) per 100 cells/mm^3^ higher, p = 0.058), being on ART (OR = 2.72 vs. being off-ART (95CI = 1.15, 6.41), p = 0.022) and calendar year of sample [OR = 0.84 (95CI = 0.77, 0.91) per more recent year, p<0.001). Baseline tropism was not associated with the risk of clinical progression or death. CD4^+^T-cell slopes did not differ within or between tropism groups.

**Conclusions:**

The predictive role of plasma tropism determined using 454 sequencing in the context of people receiving cART with detectable VL is not helpful to identify subjects at higher risk for clinical progression to AIDS or death.

## Introduction

Infection with HIV-1 with tropism for lymphocytes expressing the CXCR4 co-receptor (X4 HIV, or CXCR4-tropic HIV) [1,2] has been consistently associated with lower CD4^+^T-cell counts in antiretroviral treatment (ART)-naïve and -experienced subjects [3,4], and with faster disease progression in untreated patients [5–7], independently of their baseline CD4^+^T-cell counts or HIV-1 RNA levels [8,9]. Initially, tropism assessments were based on phenotypic assays, but such assays have limitations for clinical routine diagnostics. They are costly, time consuming, complex and require a minimum viral load of 1000 c/ml, being unsuitable for subjects with low-level viremia or those requiring CCR5 antagonists while being suppressed. Genotyping approaches using V3-loop sequencing were later implemented, being faster and cheaper [10]. Both techniques share the challenge of detecting minority virus populations that may be clinically important.

Whereas 60 to 80% of subjects at early stages of HIV infection harbour R5 HIV, X4 viruses emerge in approximately 50% of individuals at later stages of the disease [11,12]. In general, tropism switches occur from R5 to X4, but occasionally X4 to R5 switches can also be seen [13]. It has been much debated whether the emergence of X4 HIV is cause or consequence of immune depression [14]. Using modern ultrasensitive genotyping tools, X4 HIV can be detected in virtually any patient at very low levels shortly after HIV infection, suggesting that X4 HIV are being contained by the immune system. Conversely, in epidemiological studies R5 to X4 tropism switches preceded CD4+ declines [15].

Advances in genotyping techniques and the availability of bioinformatic tools to accurately predict phenotypic tropism from HIV env sequence data enable rapid, reliable and robust assessment of HIV tropism during routine clinical management. Early phenotypic tropism tools used to define the natural history of HIV, like the MT-2 or MT4 assays, were highly specific for X4 viruses but often lacked sensitivity and reproducibility across laboratories due to the difficulty in establishing cell infections. Population sequencing allows streamlined processing in low complexity labs and achieves an accuracy to predict the true tropism–defined as HIV tropism determined using the enhanced-sensitivity Trofile® assay- of around 90%, with a sensitivity to detect X4 HIV in the range of 50‐70% and specificity >90% [16]. The sensitivity and specificity of genotypic tropism prediction in plasma are improved by using massively parallel deep sequencing with either 454, Illumina or any equivalent next-generation sequencing platform [17].

Although tropism testing is mandatory before using a CCR5-inhibitor, it is uncertain whether clinicians should order a tropism test to predict if their patients could have an increased risk of clinical progression or if, instead, they should rely on other more classical predictors such as CD4+ counts, viral load etc. In a previous cohort study [11], subjects with X4 HIV had faster decline in CD4+ T-cell counts and suffered more clinical events over the following 12 months remaining off ART than those with R5 HIV. However, such differences were no longer observed in subjects receiving antiretroviral treatment.

The aim of this study was to determine whether, in people with detectable viral load, presence of X4 tropic HIV in plasma, determined using either population sequencing or 454 deep sequencing, was independently associated with an increased risk of progression to AIDS or death over the following 3 to 12 months. We also sought to evaluate in the same study population if X4 HIV was associated with steeper declines in CD4+ counts than in people with R5 HIV. The ultimate objective of our analyses was to verify whether plasma tropism testing is useful to predict clinical outcomes in routine HIV clinical management.

## Methods

### Subjects

This was a nested case-control study within the EuroSIDA cohort (described in [18]). This study underwent an Ethics Comitee in order to assure subjects confidentiality and privacy. The study complies with the regulations for Ethics Committee approval and procedure for obtaining Informed Consent from participants for the EuroSIDA Study, following the Eurosida Study Group standards. A data collection form is completed by the sites at the time of enrolment and every 6 months hereafter on to a standardized data collection form. From follow-up Winter 2014/2015 (DS41), all data collection is in the electronic CRF system, REDCap at https://chip-crf.info/redcap/. For each patient, the date of HIV diagnosis and way of transmission is recorded. The date of diagnosis of all AIDS defining diseases are recorded, using the 1993 Centers for Disease Control and Prevention definitions. Further, the date of starting and stopping each antiretroviral drug is recorded, as is the use of drugs for prophylaxis against opportunistic infections. All CD4 lymphocyte counts and viral load measurements are requested at every follow-up, as well as a range of other laboratory values. Further, data about non-AIDS defining diseases, adverse events of anti-HIV drugs (D:A:D-events), and causes of death (CoDe) are collected. In this study, cases were subjects with an AIDS diagnosis or who died from any cause, and who had an available plasma sample for tropism testing from the time window of 3 to 12 months prior to the event. In sensitivity analyses we restricted to only cases defined by AIDS or deaths due to AIDS and their matched controls. In order to minimise the risk of sequencing failure, the available sample had to have HIV-1 RNA levels above 1,000 copies/ml. Controls were subjects from EuroSIDA who also had a plasma sample available with HIV-1 RNA levels above 1,000 copies/ml and were AIDS-free and alive after a matched duration of time from the date of sampling. In the original sample scheme two controls per case were selected. Because of testing failure, some of the case-control matched set only remained with one control per case. Cases and controls were matched for age (±5 years), HIV-1 RNA levels (±0.5 log) and HCV status at the time of sampling. These matching factors were chosen because they are known to be associated with both HIV tropism and the risk of clinical progression and unlikely to be on the causal pathway between tropism and clinical progression.

### Genotypic HIV tropism testing

HIV-1 tropism was assessed using both 454 (Roche Diagnostics, Basel, Switzerland) and population sequencing of the V3-loop of gp120 as previously described [19]. Tropism testing was performed blinded for case-control status and other exposures. V3-loop sequences obtained with 454 sequencing were interpreted using the Geno2Pheno_[454]_ algorithm. In concordance with previous works [19–21] a sample was defined as containing X4 HIV if at least 2% of the sequences obtained had a Geno2Pheno false-positive rate ≤3.75%; such cutoffs showed strong association with virological outcomes in pivotal maraviroc clinical trials. Sensitivity analyses were also performed using a cut-off of at least 1% of the sequences obtained having a false positive rate of <10%, which showed the best technical correlation with Monogram®’s Enhanced-sensitivity Trofile assay [22]. Population sequencing of the V3 loop was performed using the TruGene® HIV-1 Genotyping Assay *(OpenGene DNA sequencing system*). Tropism was inferred from V3-loop population sequences using the Geno2Pheno_[co-receptor]_ tool. Samples with a Geno2Pheno false-positive rate ≤10% were considered to have X4 HIV.

### Statistical analyses

Conditional logistic regression was used to investigate the association between a number of exposure factors and the risk of clinical progression to AIDS or death. Separate logistic regression models were used for tropism estimated using 454 and population sequencing. Besides the adjustment obtained by design for the matching factors (i.e. age, HIV viral load and HCV Ab status), we further controlled for CD4^+^T-cell counts and calendar year of sample by including these factors in the regression models.

We also compared CD4 slopes in people with R5 and X4 HIV over the 12 months following the date of sampling using a linear mixed model with random intercept and slope using the whole study populations of cases and controls. The difference in CD4 slope between people carrying a X4 vs. a R5 virus was formally tested by including an interaction term in the linear model. The linear mixed model also included gender, age, HCV co-infection, current viral load, use of ART, mode of HIV transmission, nadir CD4^+^T-cell count, ethnicity and calendar year of sample. Three separate analyses were performed: the first using all CD4^+^T-cell values available per person, a second one restricted to values determined while subjects were ART-naïve, and a third analysis using only values obtained after the date of ART initiation.

## Results

### Participant characteristics

A total of 300 participants (100 cases and 200 matched controls) underwent tropism testing. Envelope amplification failed in 81 (27%) subjects for 454 sequencing, and in 104 (34%) for population sequencing (PS). In total, this left 266 evaluable subjects, 100 were cases and 166 were controls, who were tested on a sample stored on average in 2006 (IQR: 2003–2009) (Table 1). In a subset of 191 unique samples for which tropism could be estimated using both 454 (3.75% FPR) and population sequencing (10% FPR), 130 (68%) were concordantly classified as R5 and 30 (16%) as X4 for a kappa statistic of 55% (p<0.001). Globally, one quarter of patients had X4 HIV (Table 1). Our study included 20% women, 47% men who have sex with men (MSM), 92% were Caucasians and 22% had HCV co-infection. At the time of sampling, 26% were ART-naïve, 25% had begun ART but were currently off therapy and 49% were currently receiving ART. The median age, CD4^+^T-cell and HIV-1 RNA levels were 41 years, 350 cells/mm^3^ and 4.81 log c/mL, respectively. Median CD4^+^T-cell counts available per subject were 3.3 counts/year (IQR = 0.7; 8.8).

**Table 1:** Subjects’ characteristics according to 454 tropism determination.

Characteristics	X4	R5	Total	p-value
	N = 61	N = 205	N = 266	
**Age, years**				0.301
Median (IQR)	42 (37, 47)	41 (35, 49)	41 (36, 49)	
**Viral load, log10 copies/mL**				0.764
Median (IQR)	4.85 (4.45, 5.29)	4.81 (4.47, 5.38)	4.81 (4.46, 5.36)	
**Gender, n(%)**				0.135
Female	8 (13.1%)	44 (21.5%)	52 (19.5%)	
**Ethnicity, n(%)**				0.235
White	54 (89%)	190 (93%)	244 (92%)	
Asian	0 (0%)	2 (1%)	2 (1%)	
Black	1 (2%)	2 (1%)	3 (1%)	
Other/unknown	6 (10%)	11 (5%)	17 (6%)	
**Mode of HIV transmission, n(%)**				0.587
Men who have Sex with Men	28 (46%)	96 (47%)	124 (47%)	
Heterosexual transmission	6 (10%)	16 (8%)	22 (8%)	
IDU	18 (30%)	36 (18%)	54 (20%)	
Other/unknown	9 (15%)	57 (28%)	66 (25%)	
**CD4 count, cells/mm^3^**				0.348
Median (IQR)	279 (150, 471)	351 (150, 530)	350 (150, 490)	
**HCV co-infection, n(%)**				0.116
No	52 (85%)	156 (76%)	208 (78%)	
Yes	9 (15%)	49 (24%)	58 (22%)	
**Calendar year of test**				0.680
Median (IQR)	2006 (2001, 2009)	2006 (2003, 2008)	2006 (2003, 2009)	
**Clinical outcome, n(%)**				0.559
Case	21 (34%)	79 (39%)	100 (38%)	
**ART-naive, n(%)**				0.736
Yes	29 (48%)	102 (50%)	131 (49%)	

### Factors associated with clinical progression to AIDS and / or death

Table 2 shows the results from fitting a regression model with tropism estimated using 454 sequencing. The regression models using tropism estimated by population sequencing provided similar results (not shown). Exposures independently associated with risk of clinical progression to AIDS and/or to death from any cause were female gender (OR = 2.13 vs. male; 95% CI = 1.04, 4.36; p = 0.038), CD4^+^T-cell count (OR = 0.90 per 100 cells/mm^3^ higher; 95% CI = 0.80, 1.00; p = 0.058), being on ART at the time of testing (OR = 2.72 vs. being off ART; 95% CI = 1.15, 6.41; p = 0.022) and calendar year of sample (OR = 0.84 per more recent year; 95%CI = 0.77, 0.91; p<0.001). Similar predicting factors were identified when the case definition was restricted to clinical progression and/or AIDS-related deaths (Table 2). Results were similar when using a FPR of 10% (S1 Table). Factors associated with clinical progression to AIDS or AIDS-related deaths were female gender (OR = 2.77 vs. male; 95% CI = 1.16, 6.63; p = 0.022), CD4^+^T-cell count (OR = 0.83 per 100 cells/mm^3^ higher; 95% CI = 0.72, 0.96; p = 0.012), being on ART (OR = 4.66 vs. being off ART; 95% CI = 1.56, 13.89; p = 0.006) and calendar year of sample (OR = 0.84 per more recent year; 95%CI = 0.76, 0.93; p = 0.001). Baseline tropism (presence of X4 HIV), either by 454 (Table 2) or population sequencing, (S2 Table) was not associated with the risk of clinical progression or death in any of the analyses (OR = 0.66, 95% CI = 0.33, 1.33; p-value = 0.245 with the original definition of cases, or OR = 0.44, 95% CI = 0.18, 1.05; p-value = 0.064 when restricting only to deaths due to AIDS).

**Table 2:** Factors associated with risk of AIDS and/or death [a) from any cause; b) related to AIDS only] from fitting a conditional logistic regression; model using 454 to infer tropism.

	a) Association with risk of AIDS or death from any cause				b) Association with risk of AIDS or death related to AIDS only			
Factor	Case	Control	OR (95% CI)	P-value	Case	Control	OR (95% CI)	P-value
	N = 100	N = 166			N = 72	N = 120		
**Tropism (454 estimate^**c**^), n(%)**								
R5	79 (79.0%)	126 (75.9%)	1.00		59 (81.9%)	89 (74.2%)	1.00	
X4	21 (21.0%)	40 (24.1%)	0.66 (0.33, 1.33)	0.245	13 (18.1%)	31 (25.8%)	0.44 (0.18, 1.05)	0.064
**Gender, n(%)**								
Male	75 (75.0%)	139 (83.7%)	1.00		50 (69.4%)	105 (87.5%)	1.00	
Female	25 (25.0%)	27 (16.3%)	2.13 (1.04, 4.36)	0.038	22 (30.6%)	15 (12.5%)	2.77 (1.16, 6.63)	0.022
**Age^**d**^, years**								
Median (IQR)^d^	40 (35, 48)	42 (36, 50)			41 (35, 50)	42 (36, 51)		
**Viral load^**d**^, log10 copies/mL**								
Median (IQR)	4.83 (4.44, 5.25)	4.81 (4.48, 5.38)			4.80 (4.44, 5.31)	4.78 (4.48, 5.38)		
**CD4+T-cell count^**a**^, cells/mm^3^**								
Median (IQR)	285 (132, 417)	357 (201, 548)	0.90 (0.80, 1.00)	0.058	289 (141, 416)	381 (242, 559)	0.83 (0.72, 0.96)	0.012
**ART use, n(%)**								
Not started	20 (20.0%)	49 (29.5%)	1.00		11 (15.3%)	40 (33.3%)	1.00	
Started, currently on ART	31 (31.0%)	35 (21.1%)	2.72 (1.15, 6.41)	0.022	20 (27.8%)	19 (15.8%)	4.66 (1.56, 13.89)	0.006
Started, currently off ART	49 (49.0%)	82 (49.4%)	1.42 (0.65, 3.09)	0.381	41 (56.9%)	61 (50.8%)	2.67 (0.96, 7.39)	0.059
**Co-infection with HCV^**d**^, n(%)**								
No	77 (77.0%)	131 (78.9%)			64 (88.9%)	110 (91.7%)		
Yes	23 (23.0%)	35 (21.1%)			8 (11.1%)	10 (8.3%)		
**Mode of HIV transmission, n(%)**								
Homosexual contacts	42 (42.0%)	82 (49.4%)	1.00		24 (33.3%)	27 (22.5%)	1.00	
IVDU	21 (21.0%)	33 (19.9%)	1.16 (0.47, 2.90)	0.745	10 (13.9%)	17 (14.2%)	1.31 (0.40, 4.31)	0.652
Heterosexual contacts	10 (10.0%)	12 (7.2%)	0.91 (0.32, 2.58)	0.859	8 (11.1%)	10 (8.3%)	1.18 (0.36, 3.92)	0.781
Other/unknown	27 (27.0%)	39 (23.5%)	1.84 (0.86, 3.97)	0.117	24 (33.3%)	27 (22.5%)	2.54 (1.07, 6.03)	0.035
**CD4+T-cell count nadir^**a**^, n(%)**								
Median (IQR)	174 (58, 289)	202 (55, 360)	1.06 (0.84, 1.34)	0.642	201 (58, 301)	204 (56, 362)	1.21 (0.90, 1.61)	0.202
**Calendar year of sample^**b**^**								
Median (IQR)	2004 (2002, 2006)	2007 (2004, 2010)	0.84 (0.77, 0.91)	< .001	2004 (2003, 2006)	2007 (2005, 2010)	0.84 (0.76, 0.93)	0.001
**Ethnicity, n(%)**								
White	90 (90.0%)	154 (92.8%)	1.00		64 (88.9%)	109 (90.8%)	1.00	
Non white	10 (10.0%)	12 (7.2%)	1.02 (0.35, 2.92)	0.976	8 (11.1%)	11 (9.2%)	0.85 (0.26, 2.80)	0.784
**Drug resistance, n(%)**								
None	65 (65.0%)	131 (78.9%)	1.00		47 (65.3%)	96 (80.0%)	1.00	
≥1 class	35 (35.0%)	35 (21.1%)	1.31 (0.63, 2.73)	0.476	25 (34.7%)	24 (20.0%)	1.34 (0.54, 3.32)	0.527

Data adjusted for matching factors, CD4^+^T-cell count and calendar year of sampling.

^a^OR per 100 cells/mm^3^ higher.

^b^OR per more recent year.

^c^Declared X4 if ≥2%of minority population when using a 3.75% FPR.

^d^Matching factor.

### CD4^+^T-cell slopes by HIV tropism

When analyzing CD4^+^T-cell changes considering all available CD4 count values, we found no statistically significant difference in CD4^+^T-cell count slope between X4 and R5 tropism groups (p-value for interaction = 0.67). When considering only CD4^+^T-cell changes using values measured before the date of ART initiation there was a general decrease of CD4^+^T-cell count in both tropism groups but again with no difference in mean slope (-96 cells/mm^3^/year (95% CI: -325, 133) in people carrying X4 viruses vs. -158 (-283, -34) cells/mm^3^/year in the R5 group, p = 0.64, Table 3). Similarly, in the analysis using CD4^+^T-cell values measured after the date of ART initiation, as expected, CD4^+^T-cell counts showed an increase, which again was no different according to tropism (+36 cells/mm^3^/year in the X4 HIV group vs. +59 in R5 group, p = 0.67). In addition, there was no evidence that the difference in slope between X4 and R5 tropism varied by ART status (interaction p-value = 0.26), suggesting that the lack of association was consistent regardless of ART use (Table 3).

**Table 3:** Interaction between effects of tropism and ART status on CD4 slope from fitting a linear mixed model with random intercept and slope.

	X4^&^		R5		Difference X4 vs. R5	
Period	Mean CD4 count change^*^ (95% CI)	p-value	Mean CD4 count change^*^ (95% CI)	p-value	Mean CD4 count change^*^ (95% CI)	p-value
All periods	-10 (-104, 83)	0.830	11 (-46, 69)	0.701	-21 (-120, 77)	0.670
Periods before starting ART	-96 (-325, 133)	0.414	-158 (-283, -34)	0.014	63 (-198, 323)	0.638
Periods after starting ART	36 (-62, 134)	0.468	59 (-2, 119)	0.057	-23 (-125, 80)	0.666

(Interaction p-value for the difference by tropism (X4 vs. R5) between the periods before starting ART and after starting ART = 0.26).

^&^Declared X4 if ≥2% of minority population when using a 3.75% FPR, by 454.

*per year.

## Discussion

In this study we have compared the difference in risk of short-term (3–12 months) progression to AIDS/death between people carrying predominantly R5 or X4 viruses, taking into account CD4^+^T-cell count and use of ART. Plasma HIV-1 tropism, regardless of how estimated, did not predict the short-term risk of the composite outcome of AIDS or death over 3–12 months from the date of sample, after controlling for co-infection with HCV, age, current viral load, CD4 count and calendar year. CD4^+^T-cell slopes were not significantly different between tropism groups, and there was no evidence that this difference varied when separately considering treated and untreated subjects. It is important to point out that when we used an alternative case definition including only deaths due to AIDS, results were similar, although the analysis had less statistical power. We have obtained the same results using 454 sequencing and population sequencing (PS), which may indicate that the lack of association between tropism and clinical progression in our study was not due to lack of sensitivity when detecting low-frequency X4 HIV.

One limitation of the analysis of the comparison of the CD4 count slopes is the low statistical power when stratifying subjects in tropism groups and on/off ART, as in some of the sub-populations we have few subjects. Analyses took into consideration factors known to be associated with risk of AIDS progression, such as age at diagnosis, mode of HIV transmission, nadir CD4^+^T-cell count, sex and ethnicity [23–25]. Prior studies have also reported positive and negative associations between specific human leucocyte antigen (HLA) alleles and the rate of HIV-1 disease progression [26,27]. We did not evaluate the role of HLA in clinical progression in our analysis because conclusive studies regarding the influence of HLA on HIV-1 and, in general, on infectious diseases require large number of samples, ethnicity stratification, precise clinical information and consideration of other known genetic effects on the disease.

Besides the clinical binary end-point assessed in the case-control analysis we also evaluated CD4^+^T-cell count variations over time because this measure has been a central predictor of HIV disease progression and death, has been the main means to evaluate suitability for treatment initiation [28,29] and is used by the World Health Organization (WHO) to define disease staging [30]. Tropism was not associated with differences in CD4 slopes, neither before nor after starting ART.

In our analysis, tropism was measured in plasma, so patient selection was conditioned by the fact that participants had to have a VL of at least 1000 c/mL to minimize the risk of sequencing failure and having missing data for tropism. Therefore, among people who currently had a detectable viral load, we were comparing the risk of events in those who, at the time of the stored sample, had started ART with those who were still ART-naïve. The most likely explanation of our finding is the presence of confounding by indication as, for a given high viral load, ART is typically initiated in people with lower CD4 count and with a higher risk of AIDS and death. Although the estimates are adjusted for CD4^+^ T-cell count we cannot rule out the presence of residual confounding. In addition, EuroSIDA does not collect the data of HIV seroconversion and therefore the time spent with HIV is a plausible unmeasured confounder that we could not control for.

Lack of amplification was not uncommon, and this might be attributable to the highly variable sequence of the envelope gene. Another possibility is that due to the small volumes in PCR reactions it is possible that stochastically, no viral molecules were taken, as viral loads were not high in many cases. We performed analysis of the predictors of amplification failure using a logistic regression. The two independent predictors of amplification failure were HIV subtype (48% failure in non B-virus vs. 29% in B virus, p<0.0001) and lower level of viral load (mean VL 4.60 log_10_ copies/mL in amplifications failures vs. 4.78 log_10_ copies/mL in successful amplifications, p = 0.04) (S3 Table). Because viral load is also moderately associated with clinical progression it is possible that some selection bias was introduced. This is somewhat inevitable in analysis involving retrospective amplification of PCR as amplification is difficult at low levels of viremia.

For 454 sequencing we used the FPRs values recommended at the time of the analysis (e.g. 3.75% and 10%), but results were similar when using a FPR of 10%. Some retrospective re-analyses of pre-treatment plasma samples from maraviroc trials in ART-naïve and ART-experienced subjects showed that 454 sequencing and/or population sequencing for tropism inference were able to predict virological response to maraviroc as accurately as Trofile and ESTA. For Sanger sequencing, we used a FPR of <10%, which is the recommended by the guidelines, because we wanted to have results that could be applicable to clinical practice.

We defined short term as a period of 3–12 months because data from the literature show that AIDS and death in natural history appear to occur relatively quickly after the switch from a R5 to X4 virus. Therefore we designed the nested case-control analysis to evaluate the short-term risk. Also, the cross-sectional nature of the study design does not allow to control effectively for potential time-dependent confounders. Thus, by evaluating the short-term risk we wanted to try to protect the results from this other important source of bias.

Ideally, people with undetectable viral load could also be included and tropism estimated using proviral DNA. However, the number of clinical events in people with suppressed viral load would likely be very small and therefore a much larger study population would be needed to show any differences, if they existed. Moreover, the degree of clinical validation of genotypic tropism tests in clinical trials, at least in relation to response to CCR5 antagonists, is by far much stronger in plasma than in PBMCs [21].

Whether subjects with X4 HIV have impaired CD4+ T-cell count recovery during ART remains unclear. Some studies reported poorer CD4^+^T-cell count recovery in virologically suppressed ART-treated subjects with X4 HIV relative to individuals with R5 HIV [5,31]. In a large cohort study in London, subjects with dual/mixed or X4 HIV had faster CD4+ T-cell count declines than those with R5 HIV in the absence of ART. However, CD4^+^T-cell count increases, time to viral suppression and rates of viral suppression over 2-years were similar between groups in treated subjects [11]. In a study characterizing the viral and host factors influencing rapid HIV progression [32], higher virus replicative capacity and CXCR4 coreceptor usage were closely linked to faster CD4^+^T-cell depletion and rapid disease progression. In a previous analysis conducted by some of us, subjects with late presentation and <100 CD4^+^T-cell counts at HIV diagnosis who had X4 HIV by 454 sequencing at baseline, had significantly lower CD4^+^T-cell counts than those infected with an R5 HIV and showed an impaired CD4^+^T-cell counts recovery over the ensuing two years of ART [19]. Discrepancies between results in different studies may be due to the heterogeneity of the populations that are being studied (e.g. differences in viral load and CD4^+^T-cell counts at study entry, use of antiretrovirals and duration of follow-up).

We found that calendar year of sample was a factor independently associated with the risk of clinical progression or death (16% risk reduction per more recent year). This may be explained due to the improvements in the clinical management of the patients, differences in the actual drugs used, recent regimens typically being more efficacious with a better toxicity profile and easier to take than in the past.

Being a woman in this population also meant to have increased risk of progression to AIDS or death (almost a 3–fold increased risk compared to men). It has been previously reported from studies in Europe that women have higher rates of virological failure and increased rates of discontinuation of treatment [33,34]. Of note, however, the effect of gender in our study was independent of HIV-1 RNA and CD4 count. Women tend to be HIV-diagnosed later than men who have sex with men (MSM) [35], which likely reflects barriers on access to care. Additionally, in the UK, for example, HIV infected women have different origins, ethnicities and socioeconomic status, compared to HIV positive men[36]. Another factor independently associated with the risk of progression was current use of ART. Although the OR might appear to go in the opposite direction of that expected (higher risk in treated vs. not treated) this could be explained by confounding by indication, given the conducted analysis, as those subjects on ART and with a viral load ≥1000 copies/ml are actually going towards a virological failure.

In conclusion, our results suggest that the predictive role of plasma tropism determined using 454 sequencing in the context of people receiving cART with detectable VL is not helpful to identify subjects at higher risk for clinical progression after controlling for age, plasma HIV viral load and HCV serostatus. Clinicians should rather consider gender, CD4+ counts and virological outcomes of ART for that purpose. Further studies are needed to evaluate the role of tropism testing in proviral DNA to predict clinical progression to AIDS and/or death.

## Supporting Information

S1 Table Factors associated with risk of AIDS and/or death from any cause from 454 sequencing using a FPR of 10%. (DOCX)

S2 Table Factors associated with risk of AIDS and/or death from fitting a conditional logistic regression; model using Sanger sequencing to infer tropism. (DOCX)

S3 Table Logistic regression analyses of factors associated to amplification failure. (DOCX)
