# Supplementary material for: Plasma HIV-1 Tropism and the Risk of Short-Term Clinical Progression to AIDS or Death
Source: PLoS One. 2017 Jan 27;12(1):e0166613. doi: 10.1371/journal.pone.0166613 (PMC5271314; doi:10.1371/journal.pone.0166613)
Supplement: S1 Table — (DOCX) [file pone.0166613.s001.docx]

**S1 Table. Factors associated with risk of AIDS and/or death from any cause from 454 sequencing using a FPR of 10%.**

| **Association with risk of AIDS/death from fitting a conditional logistic regression** | | | | | | | | | | |
| --- | --- | --- | --- | --- | --- | --- | --- | --- | --- | --- |
|  | **Event** | **Event free** | **Adjustment(1)** | | **Adjustment(2)** | | **Adjustment(3)** | | **Adjustment(4)** | |
| **Factor** | N= 100 | N= 166 | **OR^1^ (95% CI)** | **P-value** | **OR^2^ (95% CI)** | **P-value** | **OR^3^ (95% CI)** | **P-value** | **OR^4^ (95% CI)** | **P-value** |
| **Tropism (454 estimate^+^), n(%)** |  |  |  |  |  |  |  |  |  |  |
| R5 | 44 (44.0%) | 63 (38.0%) | 1.00 |  | 1.00 |  | 1.00 |  | 1.00 |  |
| X4 | 56 (56.0%) | 103 (62.0%) | 0.78 (0.47, 1.29) | 0.330 | 0.75 (0.44, 1.28) | 0.293 | 0.76 (0.45, 1.26) | 0.284 | 0.72 (0.42, 1.23) | 0.232 |
| **Gender, n(%)** |  |  |  |  |  |  |  |  |  |  |
| Male | 75 (75.0%) | 139 (83.7%) | 1.00 |  | 1.00 |  | 1.00 |  | 1.00 |  |
| Female | 25 (25.0%) | 27 (16.3%) | 1.72 (0.93, 3.17) | 0.084 | 2.05 (1.06, 3.96) | 0.033 | 1.45 (0.77, 2.73) | 0.250 | 1.84 (0.94, 3.62) | 0.076 |
| **Age^++^, years** |  |  |  |  |  |  |  |  |  |  |
| Median (IQR)^++^ | 40 (35, 48) | 42 (36, 50) |  |  |  |  |  |  |  |  |
| **Viral load^++^, log10 copies/mL** |  |  |  |  |  |  |  |  |  |  |
| Median (IQR) | 4.83 (4.44, 5.25) | 4.81 (4.48, 5.38) |  |  |  |  |  |  |  |  |
| **CD4 count^*^, cells/mm3** |  |  |  |  |  |  |  |  |  |  |
| Median (IQR) | 285 (132, 417) | 357 (201, 548) | 0.85 (0.76, 0.95) | 0.004 | 0.89 (0.80, 1.00) | 0.060 |  |  | 0.89 (0.80, 1.00) | 0.060 |
| **ART use, n(%)** |  |  |  |  |  |  |  |  |  |  |
| Not started | 20 (20.0%) | 49 (29.5%) | 1.00 |  | 1.00 |  | 1.00 |  | 1.00 |  |
| Started, currently on ART | 31 (31.0%) | 35 (21.1%) | 2.17 (1.07, 4.41) | 0.032 | 1.86 (0.88, 3.94) | 0.106 | 1.89 (0.92, 3.91) | 0.085 | 1.74 (0.81, 3.73) | 0.152 |
| Started, currently off ART | 49 (49.0%) | 82 (49.4%) | 1.46 (0.78, 2.75) | 0.235 | 1.53 (0.78, 3.00) | 0.212 | 1.09 (0.56, 2.12) | 0.808 | 1.29 (0.64, 2.60) | 0.476 |
| **Co-infection with HCV^++^, n(%)** |  |  |  |  |  |  |  |  |  |  |
| No | 77 (77.0%) | 131 (78.9%) |  |  |  |  |  |  |  |  |
| Yes | 23 (23.0%) | 35 (21.1%) |  |  |  |  |  |  |  |  |
| **Mode of HIV transmission, n(%)** |  |  |  |  |  |  |  |  |  |  |
| Homosexual contacts | 27 (27.0%) | 39 (23.5%) | 1.00 |  | 1.00 |  | 1.00 |  | 1.00 |  |
| IVDU | 21 (21.0%) | 33 (19.9%) | 1.24 (0.64, 2.41) | 0.520 | 0.88 (0.43, 1.80) | 0.720 | 1.07 (0.54, 2.11) | 0.842 | 0.81 (0.39, 1.67) | 0.562 |
| Heterosexual contacts | 10 (10.0%) | 12 (7.2%) | 1.63 (0.65, 4.07) | 0.299 | 0.90 (0.33, 2.46) | 0.842 | 1.44 (0.57, 3.66) | 0.444 | 0.85 (0.31, 2.35) | 0.753 |
| Other/unknown | 27 (27.0%) | 39 (23.5%) | 1.35 (0.73, 2.50) | 0.337 | 1.45 (0.75, 2.78) | 0.267 | 1.20 (0.64, 2.25) | 0.578 | 1.34 (0.69, 2.59) | 0.388 |
| **CD4 count nadir^*^, n(%)** |  |  |  |  |  |  |  |  |  |  |
| Median (IQR) | 174 (58, 289) | 202 (55, 360) | 0.87 (0.75, 1.01) | 0.069 | 0.94 (0.80, 1.10) | 0.427 | 1.06 (0.85, 1.31) | 0.621 |  |  |
| **Calendar year of sample^**^** |  |  |  |  |  |  |  |  |  |  |
| Median (IQR) | 2004 (2002, 2006) | 2007 (2004, 2010) | 0.83 (0.77, 0.89) | <.001 |  |  | 0.84 (0.78, 0.91) | <.001 | 0.84 (0.78, 0.91) | <.001 |
| **Ethnicity, n(%)** |  |  |  |  |  |  |  |  |  |  |
| White | 90 (90.0%) | 154 (92.8%) | 1.00 |  | 1.00 |  | 1.00 |  |  |  |
| Non white | 10 (10.0%) | 12 (7.2%) | 1.43 (0.59, 3.43) | 0.429 | 1.28 (0.49, 3.35) | 0.609 | 1.52 (0.62, 3.73) | 0.360 |  |  |
| **Drug resistance, n(%)** |  |  |  |  |  |  |  |  |  |  |
| None | 65 (65.0%) | 131 (78.9%) | 1.00 |  | 1.00 |  | 1.00 |  |  |  |
| >=1 class | 35 (35.0%) | 35 (21.1%) | 2.02 (1.16, 3.51) | 0.013 | 1.43 (0.79, 2.59) | 0.240 | 1.76 (0.99, 3.12) | 0.054 |  |  |
| ^1^Adjusted, by design, for matching factors only | | | | | | | | | | |
| ^2^Adjusted for matching factors and calendar year | | | | | | | | | | |
| ^3^Adjusted for matching factors and CD4 count | | | | | | | | | | |
| ^4^Adjusted for matching factors and CD4 count and calendar year | | | | | | | | | | |
| ^*^OR per 100 cells/mm3 higher | | | | | | | | | | |
| ^**^OR per more recent year | | | | | | | | | | |
| ^+++^Declared X4 if >=1% of minority population when using a 10% FPR | | | | | | | | | | |
| ^++^Matching factor | | | | | | | | | | |
