# Supplementary material for: Plasma HIV-1 Tropism and the Risk of Short-Term Clinical Progression to AIDS or Death
Source: PLoS One. 2017 Jan 27;12(1):e0166613. doi: 10.1371/journal.pone.0166613 (PMC5271314; doi:10.1371/journal.pone.0166613)
Supplement: S2 Table — (DOCX) [file pone.0166613.s002.docx]

**S2 Table) Factors associated with risk of AIDS and/or death from fitting a conditional logistic regression; model using Sanger sequencing to infer tropism**

| **Association with risk of AIDS/death from fitting a conditional logistic regression** | | | | |
| --- | --- | --- | --- | --- |
|  | **Event** | **Event free** | **Adjustment** | |
| **Factor** | N= 86 | N= 142 | **OR (95% CI)** | **P-value** |
| **Tropism (Sanger estimate^+^), n(%)** |  |  |  |  |
| R5 | 66 (76.7%) | 107 (75.4%) | 1.00 |  |
| X4 | 20 (23.3%) | 35 (24.6%) | 1.06 (0.49, 2.29) | 0.890 |
| **Gender, n(%)** |  |  |  |  |
| Male | 68 (79.1%) | 118 (83.1%) | 1.00 |  |
| Female | 18 (20.9%) | 24 (16.9%) | 1.83 (0.80, 4.20) | 0.155 |
| **Age^++^, years** |  |  |  |  |
| Median (IQR)^++^ | 40 (35, 49) | 42 (35, 50) |  |  |
| **Viral load^++^, log10 copies/mL** |  |  |  |  |
| Median (IQR) | 4.81 (4.44, 5.28) | 4.84 (4.52, 5.38) |  |  |
| **CD4 count^*^, cells/mm3** |  |  |  |  |
| Median (IQR) | 308 (166, 417) | 366 (236, 548) | 0.90 (0.79, 1.02) | 0.108 |
| **ART use, n(%)** |  |  |  |  |
| Not started | 16 (18.6%) | 45 (31.7%) | 1.00 |  |
| Started, currently on ART | 29 (33.7%) | 29 (20.4%) | 3.13 (1.14, 8.60) | 0.027 |
| Started, currently off ART | 41 (47.7%) | 68 (47.9%) | 1.52 (0.60, 3.84) | 0.377 |
| **Co-infection with HCV^++^, n(%)** |  |  |  |  |
| No | 68 (79.1%) | 110 (77.5%) |  |  |
| Yes | 18 (20.9%) | 32 (22.5%) |  |  |
| **Mode of HIV transmission, n(%)** |  |  |  |  |
| Homosexual contacts | 21 (24.4%) | 30 (21.1%) | 1.00 |  |
| IVDU | 17 (19.8%) | 29 (20.4%) | 1.18 (0.43, 3.28) | 0.747 |
| Heterosexual contacts | 9 (10.5%) | 12 (8.5%) | 0.78 (0.26, 2.41) | 0.672 |
| Other/unknown | 21 (24.4%) | 30 (21.1%) | 1.56 (0.65, 3.73) | 0.320 |
| **CD4 count nadir, n(%)** |  |  |  |  |
| Median (IQR) | 198 (62, 304) | 206 (55, 374) | 1.13 (0.85, 1.49) | 0.408 |
| **Calendar year of sample^**^** |  |  |  |  |
| Median (IQR) | 2004 (2002, 2006) | 2007 (2004, 2009) | 0.81 (0.72, 0.90) | <.001 |
| **Ethnicity, n(%)** |  |  |  |  |
| White | 77 (89.5%) | 134 (94.4%) | 1.00 |  |
| Non white | 9 (10.5%) | 8 (5.6%) | 1.75 (0.47, 6.44) | 0.402 |
| **Drug resistance, n(%)** |  |  |  |  |
| None | 59 (68.6%) | 112 (78.9%) | 1.00 |  |
| ≥1 class | 27 (31.4%) | 30 (21.1%) | 1.39 (0.52, 3.71) | 0.505 |

Adjusted for matching factors, CD4 counts and calendar year

^*^OR per 100 cells/mm^3^ higher

^**^OR per more recent year

^+^Declared X4 using Sanger sequencing if FPR ≤10%

^++^Matching factor
