# Supplementary material for: Plasma HIV-1 Tropism and the Risk of Short-Term Clinical Progression to AIDS or Death
Source: PLoS One. 2017 Jan 27;12(1):e0166613. doi: 10.1371/journal.pone.0166613 (PMC5271314; doi:10.1371/journal.pone.0166613)
Supplement: S3 Table — (DOCX) [file pone.0166613.s003.docx]

**S3 Table) Logistic regression analyses of factors associated to amplification failure.**

|  | **Odds ratios of amplification failure** | | | |
| --- | --- | --- | --- | --- |
| **Characteristic at date of sample** | **Unadjusted OR (95% CI)** | **p-value** | **Adjusted^*^ OR (95% CI)** | **p-value** |
| ***Gender, n(%)*** |  |  |  |  |
| Female vs. male | 1.19 (0.74, 1.94) | 0.473 | 0.87 (0.48, 1.56) | 0.642 |
| ***Mode of HIV Transmission, n(%)*** |  |  |  |  |
| MSM | 1.00 |  |  |  |
| PWID | 1.51 (0.90, 2.55) | 0.118 | 1.47 (0.82, 2.63) | 0.192 |
| Heterosexual contacts | 1.72 (1.08, 2.76) | 0.023 | 1.57 (0.90, 2.74) | 0.115 |
| Other/Unknown | 0.56 (0.23, 1.36) | 0.198 | 0.53 (0.20, 1.35) | 0.181 |
| ***Ethnicity, n(%)*** |  |  |  |  |
| White vs. non-white | 1.21 (0.58, 2.52) | 0.607 | 1.72 (0.76, 3.87) | 0.194 |
| ***Subtype, n(%)*** |  |  |  |  |
| non-B vs. B | 2.31 (1.54, 3.46) | <.001 | 2.12 (1.35, 3.32) | 0.001 |
| ***Age, years*** |  |  |  |  |
| per 10 years older | 0.91 (0.76, 1.09) | 0.324 | 0.97 (0.80, 1.19) | 0.773 |
| ***Viral load, log10 copies/mL*** |  |  |  |  |
| per log lower | 1.31 (1.02, 1.68) | 0.036 | 1.38 (1.06, 1.79) | 0.015 |
| ***Calendar year*** |  |  |  |  |
| per more recent | 1.05 (0.99, 1.10) | 0.086 | 1.01 (0.95, 1.07) | 0.753 |
